# Editorial: Immigrant's health in different migration contexts

**DOI:** 10.3389/fpubh.2023.1188061

**Published:** 2023-07-13

**Authors:** Silvia Candela, Luigi Bisanti, Cristina Canova, Nicola Caranci, Alessio Petrelli

**Affiliations:** ^1^Istituto di Ricovero e Cura a Carattere Scientifico (IRCCS) Local Health Authority of Reggio Emilia, Reggio Emilia, Italy; ^2^Private Researcher, Milan, Italy; ^3^Unit of Biostatistics, Epidemiology and Public Health, Department of Cardio-Thoraco-Vascular Sciences and Public Health, University of Padova, Padua, Italy; ^4^Department of Innovation in Health and Social Care, General Directorate of Health and Welfare, Bologna, Italy; ^5^Epidemiology Unit, National Institute for Health, Migration and Poverty (INMP), Rome, Italy

**Keywords:** migration contexts, migrants on the move, undocumented migrants, resident migrants, migrants' health

Migrations are a phenomenon that dates to the mists of time, changing characteristics and intensity according to each historical moment. In the last decades it has sharply increased, as reported by the IOM 2022 World Migration Report, which states that 281 million people were living in displacement by the end of 2022 compared to 173 million in 2000 (1), and the increase is also evident in comparing the figure with the previous year (272 million), despite the mobility restrictions imposed by the SARS-CoV 2 pandemic in many countries. Many determinants have contributed to triggering this phenomenon: wars, political religious and racial persecutions, climate change that causes destruction of the territory and famine, desire for economic improvement.

The composition of the migrant population changes according to the drive to emigrate and therefore to the country of origin and the historical moment. Migrant health is a relevant indicator of the general characteristics of people moving or residing in the host country and provides crucial information on their primary needs and main healthcare needs. The migrant flows are well-monitored by international agencies (2) but their health must be the object of periodic and specific research, to provide organizers and policy-makers with the essential information on what is needed to support these people in critical moments of their life such as the displacement from the native country and the evolution of the integration process in a new country.

Migrants' health is conditioned by the characteristics of the migrant subject (age, sex, socio-economic status, and cultural background), the country of origin (conflicts, poorness, and health services organization) as well as the conditions of the migration journey and the measures of care taken in transit or arrival places (3).

This is the reason why Frontiers on Public Health launched a call for original articles on the Research Topic of migrant health in different migration contexts: resident immigrants, undocumented migrants, and migrants on the move to their final destination. The context itself identifies different typologies of migrant populations and needs, as well as different health monitoring and care measures, expected to be taken at each place. The description of the different populations offers a better understanding of the migration phenomenon as a whole, and the evaluation of such measures could provide elements of analysis, useful to plan future assistance.

The call has produced five articles, two of which relate to different health problems of resident immigrants in Italy (Ottone et al.; Dalla Zuanna et al.), one describes the monitoring of infectious diseases among undocumented migrants in France (Vignier et al.), while the two last refer to migrations recorded in other continents: French Guiana in South America (Alcouffe et al.) and South Korea in Asia (Kang et al.), where the authors refer respectively to the sexual vulnerability of migrant women and the self-perceived health among immigrant workers. A broad spectrum of situations is covered by the above-mentioned articles: can we be satisfied with the research produced? Yes and no.

Indeed, the articles provide information on different countries and approaches, and this confirms the great variety of aspects the term “migrant,” generically used, hides in itself. Thus, the health of regular residents in Italy presents aspects similar to those of the native population, mainly among chronic patients followed by the health services, confirming in this way that integration in the host country is the best way to ensure good health and lesser expenses for the health organizations over the long term. The case is different for the undocumented migrants described in France, who live in more precarious situations and have less access to health services. Although generally young, their health conditions would need “early access to care and inclusive medical psychosocial management,” as the authors conclude. Even more different is the case of women migrants to French Guiana, arriving from neighboring countries: they present some differences in behavior according to the country of origin, but many of them are substantially subject to violence and rape (“Migrant women in French Guiana are in a situation of sexual vulnerability…[and] better prevention and support for transactional sex is needed to prevent violence and its mental health and alcohol misuse consequences for all women”). Again, unmet healthcare needs are one of the main causes of poor self-reported health among immigrant workers in Korea, together with working conditions and living environments. Once more poor integration, i.e., poor access to health services and discrimination, is the source of ill health, through pathways pertaining to lifestyles, depression and loneliness. This is authoritatively confirmed by the Lancet Commission on Migration and Health, which in one of its key messages strongly reminds that:

“Universal and equitable access to health services and to all determinants of the highest attainable standard of health within the scope of universal health coverage needs to be provided by governments to migrant populations, regardless of age, gender, or legal status” (4).

The reasons for relative unsatisfaction with the texts published do not lie in the content of the papers and do not depend on the authors themselves, but it is about the awareness that in migrant health much more still needs to be studied and communicated. We need more coordinated research among the different subjects to explore new fields in describing and monitoring health inequalities by migrant status, mainly among undocumented migrants and people on their migration journey, and to develop research methods suitable for approaching such a challenging subject. We need also to find timely and effective ways to present the main results to administrators and politicians, so that research influences the decisions to be made. In fact, like all other subjects of social epidemiology, migrant health is a useful field of research only if we are aware of its social and political relevance, and develop our research consequentially.

## Author contributions

SC coordinated the editing work. SC, LB, NC, CC, and AP did the editing work. All authors contributed to the article and approved the submitted version.
